# Production, immunogenicity, stability, and safety of a vaccine against *Clostridium perfringens* beta toxins

**DOI:** 10.14202/vetworld.2020.1517-1523

**Published:** 2020-08-07

**Authors:** Mohamed J. Saadh, Issam J. Sa’adeh, Moeen F. Dababneh, Ammar M. Almaaytah, Mohammad F. Bayan

**Affiliations:** 1 Department of Pharmacy, Faculty of Pharmacy, Middle East University, Amman, Jordan; 2 Department of Radiology, King Abdulaziz Medical City, National Guard Hospital, Riyadh, Saudi Arabia; 3 Department of Pharmaceutical Technology, Faculty of Pharmacy, Jordan University of Science and Technology, Irbid, Jordan; 4 Department of Pharmaceutical Sciences, Faculty of Pharmacy, Philadelphia University, Amman, Jordan

**Keywords:** beta toxin, cattle, *Clostridium perfringens* type C, potency, safety, stability, toxoid

## Abstract

**Background and Aim:**

The beta toxin is causing the most severe *Clostridium perfringens*-related diseases. This work was dedicated to developing a vaccine against beta toxin using *C. perfringens* type C (NCTC 3180).

**Materials and Methods:**

The crude toxoid harvest contained 710 limits of flocculation (Lf)/mL. The vaccine was formulated. Each 1 mL of the final vaccine product contained at least 50 Lf/mL of beta toxoids, 0.2 mL 3% aluminum hydroxide gel (equivalent to 5.18 mg of aluminum), <0.001% W/V thiomersal, formaldehyde <0.05% W/V, and ~0.7 mL phosphate-buffered saline (pH 7.2). The efficacy of the vaccine was evaluated by potency, stability, and safety tests.

**Results:**

The vaccine demonstrated 24.36 IU/mL (standard deviation, ±0.56) and 14.74 IU/mL (±0.36) of neutralizing antibodies in rabbits and cattle, respectively. Indeed, these levels were above the minimum recommended by international protocols since the obtained antibody levels had 2.43- and 1.47-fold increase in both rabbits and cattle, respectively, over the minimum antitoxin level suggested by the United States Department of Agriculture. Interestingly, our formulation was capable of inducing 1.65-fold higher immune responses in rabbits than that stimulated in cattle (65% increase) with a significant difference (p<0.0001). The vaccine was stable up to 30 months. The vaccinated rabbits were suffered from a temporarily slight increase in temperatures in the first 10 h without any significant difference (p>0.05).

**Conclusion:**

The research showed a procedure for the manufacturing process of the vaccine against *C. perfringens* beta toxins with a feasible quantity and the vaccine described here showed to be effective in eliciting levels of neutralizing antibodies higher than required by international standards. In addition, The vaccine was stable up to 30 months. Thus, it may represent an effective and safe for preventing *C. perfringens*-related diseases in rabbits and cattle, although further studies to prove its efficacy in the field on other farm animals are still needed.

## Introduction

*Clostridium perfringens* is a spore-forming anaerobic bacterium, which is highly pathogenic for both men and animals [1-3]. *C. perfringens* is a Gram-positive omnipresent bacterium that can be found in the environment, particularly in soil and water [4]. The organism produces various toxins and enzymes that are responsible for the severe myonecrotic lesions, sometimes accompanying infections [5]. Indeed, *C. perfringens* is one of the most pathogenic species of the *Clostridium* genus and capable of producing at least 17 toxins [4]. Overall, *C. perfringens* is classified into five toxinotypes (A, B, C, D, and E) based on the gene expression of four major toxins: Alpha, beta, epsilon, and iota toxin. Besides expressing one or more of these toxinotypes, *C. perfringens* strains can produce additional toxins, including, but not limited to, enterotoxin and necrotic enteritis B-like toxin (NetB) [6]. It is worth noting that only the aforementioned four toxins are considered to be the most important since they are related to the pathogenesis of most of the *C. perfringens*-related diseases in humans and mammals. Interestingly, types B and C strains of *C. perfringens* are most commonly found throughout the environment producing beta toxin [7,8]. The molecular mass of a mature beta toxin is 35 kDa [9], and beta toxin is a trypsin-sensitive toxin causing necrotic enteritis in pigs, sheep, goats, cattle, and chickens [10]. This disease most often occurs in young animals of these species and may, in general, cause serious injury to the intestinal epithelium of both animals and humans [11,12]. Administered intravenously into rats, beta toxin causes a rise in blood pressure and a decrease in heart rate [13,14]. Remarkably, this toxin is capable of inducing the release of catecholamines, which are responsible for the increased blood pressure [13,14].

In general, the eradication of the diseases caused by *C. perfringens* toxins is almost impossible; therefore, vaccination is critical to controlling *C. perfringens*-associated diseases. There is no doubt that vaccination is considered the most effective medical intervention ever introduced, eliminating a large part of the infectious diseases once killing millions of animals and humans [15]. Therefore, this study is concerned with the production of a feasible quantity of the major *C. perfringens* beta toxin under ideal conditions and then developing a vaccine against it so as to help improve coverage and access to immunization. Moreover, this work is dedicated to estimating whether there is an impact on eliciting an immunological response in both rabbits and cattle and, if so, to what extent it could be affected compared with the recommended minimum antibody levels.

Furthermore, this study aims to examine the stability and safety of a vaccine against *C. perfringens* beta toxin through inoculating different groups of rabbits and cattle with different doses of vaccine and observing their body temperatures to exclude any local or general reaction after vaccination.

## Materials and Methods

### Ethical approval

All animal experiments were performed in accordance with the guidelines of the National Council for Animal Experimentation Control, and the Ethical Committee approval was obtained from Ethical Committee of Middle East University-Jordan.

### Study period and location

This study was conducted from March 2016 to May 2019 in Jordan Bio-Industries Center (JOVAC), Amman, Jordan.

### Sample

The manufacturing process of the final product in this manuscript was carried out using strains of *C. perfringens* type C (NCTC 3180).

### Preparation of culture medium

The culture medium used in the fermenter was prepared by dissolving its components in 1 L of distilled water. These components included 10 g meat extract, 10 g bacteriological peptone, 3 g yeast extract, 5 g D glucose, 1 g starch, 5 g sodium chloride, 3 g sodium acetate, and 0.1 g sodium bisulfite. The medium was autoclaved for 15 min at 121°C and left to cool at 25°C (pH = 6.6–7.0).

### Growth conditions of C. perfringens Type C for the production of toxins used to produce veterinary vaccines

The freeze-dried working seed vial (1×10^9^-3×10^9^ colony-forming units [CFU]) was suspended with 2 mL of the final medium and subsequently underwent serial dilution. The incubation was then carried out at 37°C under stringent anaerobic conditions for 22 h. A subculture was then performed in 75 mL cultured medium in a blue cap bottle and used as inoculum in fermenters of 20 L in the last step of the production. During fermentation, oxygen-free nitrogen was insufflated to maintain anaerobic conditions and a pH of 7.2-7.4 by adding 40% NaOH, with constant stirring at 105 rpm.

### Inactivation

Maximum bacterial growth was reached after an incubation period for 22 h at 37°C. The *C. perfringens* type C strain was inactivated by adding 5 mL commercial formaldehyde (35%-40%) to yield a final concentration in the culture of 0.2% per 1 L of culture in the fermenter with continuous agitation at 33°C for 2 days. If needed, sodium bisulfite 20% was added after inactivation to neutralize remaining formaldehyde.

### Vaccine formulation

The inactivated *C. perfringens* type C culture was clarified by centrifugation at 20,000 rpm (we took a sample before inactivation for limit of flocculation [Lf] testing). From the supernatant, 81 mL (710 Lf/mL) was taken and diluted by adding 718 mL of phosphate-buffered saline (PBS) (pH=7.2). Thiomersal was ultimately added to obtain the final concentration (0.001% W/V). In addition, 200 mL 3% aluminum hydroxide gel was added to 1 L of crude harvest (20% V/V) and agitated continuously for about 1 h; the pH was finally adjusted to 6.8–7.2. Each milliliter of the final product (*C. perfringens* type C vaccine) contained beta toxoids commensurate to 57 Lf/mL (final product must contain at least 50 Lf; however, we included 15% extra), 0.2 mL 3% aluminum hydroxide gel (equivalent to 5.18 mg of aluminum), <0.001% W/V thiomersal, formaldehyde <0.05% W/V, and ~0.7 mL PBS (pH=7.2).

### Titration quantification test for *C. perfringens* Type C

A total of 0.5 mL of *C. perfringens* type C culture was added to 4.5 mL PBS (pH=7.2) to obtain a dilution of 10^−1^, which was subsequently diluted serially using PBS for obtaining 10^−2^, 10^−3^, 10^−4^, 10^−5^, 10^−6^, and 10^−7^ dilutions, and so on, until reaching the desired dilution according to the titer of bacteria in CFU/mL. Next, a total of 0.1 mL of 10^−6^ and 10^−7^ dilutions were inoculated separately onto three Reinforced Clostridial Agar plates. These plates were then incubated at 37°C for 3-4 days, and the number of colonies on each plate was counted. Finally, the mean values of these counted colonies were calculated and multiplied by the dilution factor so as to obtain the final titer in CFU/mL.

### Inactivation kinetics test of the culture

Formaldehyde was used as an inactivating agent. The inactivation process was checked every 4 h on the 1^st^ day and later every 8 h to monitor the total inactivation time. A total of 20 mL of each sample was centrifuged for approximately 15 min at 16,000 rpm. The supernatant was then discarded, whereas the pellet was reconstituted with sterile peptone water. This step was repeated 3 times to eliminate residual formaldehyde, and thereafter, the aforementioned quantification test was performed.

### Inactivation kinetics test of beta toxin

A random sample of 100 mL was taken every 12 h during the first 48 h and later every 24 h, maintaining the culture temperature at 37°C. Every sample was centrifuged for 15 min at 16,000 rpm, and the supernatant, which included the toxin, was filtrated through a 0.45 μm filter and subsequently through a 0.2 μm filter. To eliminate the residual formaldehyde from the supernatant, the samples underwent several consecutive concentrations and dilutions with normal saline solution. Toxigenesis was determined by estimating LD_50_/mL (Lf/mL) for every sample. The samples were diluted serially using normal saline as follows: 1:100, 1:200, 1:400, 1:800, and 1:1600. Next, five healthy mice weighing 18-22 g were intravenously injected with 0.25 mL of each dilution according to the European Pharmacopoeia [16]. The mice were then kept under observation for 7 days, and toxin titers were calculated in LD_50_/mL (Lf/mL) by the Spearman–Karber method [17].

### Determination of the Lf

This test was carried out for the *C. perfringens* type C culture before the inactivation and after the inactivation process so as to determine the beta toxin in Lf. One milliliter of standard National Institute for Biological Standards and Control (NIBSC) beta antitoxin containing 100 Lf/mL was diluted using normal saline (1:1, 1:2, 1:3, 1:4, 1:5, 1:6, 1:7, 1:8, 1:9, and 1:10), and the volume was made up to 1 mL. Next, 1 mL beta toxin was diluted 1:40 using normal saline. The beta toxin (1:40) was then added to each tube, and the solution was mixed by shaking. The tubes were then placed in a water bath at 56°C. The tubes were continuously monitored. The first tube showing flocculation contained the optimum amount of antitoxin neutralizing the toxin. The Lf for the toxin was calculated from the concentration of antitoxin in the first tube, showing flocculation [18].

### Potency test in rabbits

The potency test was performed using 20 female New Zealand rabbits aged 3-6 months and weighing approximately 1.5 kg. The rabbits were categorized into two groups, with ten rabbits in each. Group 1 received the formulated vaccine, while the other group (negative control) received only sterile PBS mixed with aluminum hydroxide (Al(OH)_3_). In addition, five cattle were also included in the potency test and received the formulated vaccine as Group 1, while the other group (negative control) received only sterile PBS mixed with aluminum hydroxide. The potency test was repeated every 3 months up to 12 months and every 6 months up to 30 months to study vaccine stability.

Rabbits (n=10) and cattle (n=5) were subcutaneously immunized with 1 mL and 3 mL, respectively, on days 0 and 21, and blood was collected on day 35 for rabbits. Cattle were immunized on days 0 and 42, and blood was drawn on day 72. Whole blood was collected for each group in a sterile tube, which was then allowed to clot at room temperature to obtain sera through centrifugation. The sera obtained from each group were pooled, resulting in one pool of serum per group [19-21], which was then used in the seroneutralization assay.

### Seroneutralization assay

For measuring beta antitoxins in international units per mL (IU/mL), 1 mL of each standard toxin (NIBSC) was incubated at 37°C for 1 h with 1 mL of each pooled sera in serial dilutions from 1:1 to 1:32. Next, ten mice weighing 18-22 g were intravenously inoculated with 0.2 mL of each sample and subsequently observed for 72 h for survival and then euthanized if necessary. The procedure was repeated with intermediary dilutions of the sera to identify the lowest protective dilution. The procedures were based on the British Pharmacopoeia United States Department of Agriculture (USDA) [21], British Pharmacopeia and European Pharmacopoeia [19,20,22], and Code of Federal Regulations Title 9 (CFR 9, USA) [19] for measuring beta antitoxins.

### Safety

Twenty rabbits (15 young rabbits and five adult rabbits) were used in the safety test. These rabbits were free of specific antibodies toward the formulated vaccine antigen. Both young and adult rabbits were inoculated with the final product of our developed vaccine, and rectal temperature was measured to examine if the vaccine was safe and not causing any local or general reactions or adverse effects in the vaccinated animals, regardless of age. Overall, the included rabbits were categorized into four main groups according to the administered dose. The first group included five young rabbits that were vaccinated with only one dose of 1 mL for each animal. The second group included five young rabbits that were vaccinated with a double dose. The third group included five young rabbits that were vaccinated twice; the time interval between the first and second dose was 21 days. Finally, the fourth group included five adult rabbits vaccinated with one dose [23].

### Statistical analysis

Statistical analyses were performed using the Statistical Package for the Social Sciences (SPSS) software v.15.0 (SPSS Inc., Chicago, IL) and the GraphPad Prism package v.5.0 (GraphPad Software, San Diego, CA). Continuous variables were expressed as means ± SD. Statistically significant differences between groups were determined using ANOVA and Student’s t-test. A probability p>0.05 was considered non-significant, p<0.05 was considered significant, and p<0.0001 was considered extremely significant.

## Results

**Figure-1:**
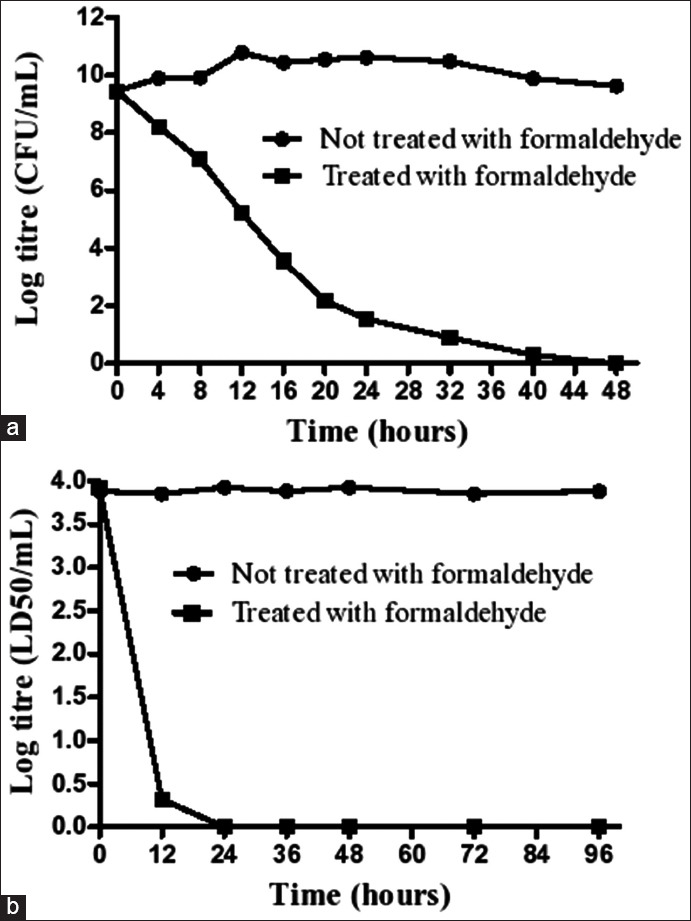
Inactivation kinetic of (a) *Clostridium perfringens* type C, and (b) its respective beta toxin in comparison with those without any inactivation. Formaldehyde was used as an inactivating agent in producing vaccines. The inactivation process of *C. perfringens* type C was checked every 4 h on the 1^st^ day and later every 8 h to check the total inactivation time. The inactivation process of beta toxin was checked every 12 h during the first 48 h and later every 24 h.

The inactivation kinetics for *Clostridium perfringens* Type *C* after cultivation for 24 h as shown in Table-1, to monitor the time of total inactivation. Interestingly, *C. perfringens* type C was found to be completely inactivated after 2 days (48 h), as shown in Figure-1a. It is noteworthy that the aforementioned process was mandatory to eliminate any possible harmful effects and destroys the pathogen´s ability to replicate. Toxigenesis was then determined by estimating the median lethal dose (LD_50_ Lf/mL) for different samples (100 mL each) and obtained every 12 h, as summarized in Table-2. Aliquots of the various toxin dilutions (1:100, 1:200, 1:400, 1:800, and 1:1600) were inoculated subcutaneously into five healthy mice weighing 18-22 g so as to check the time of total inactivation. The toxin was completely converted into toxoid (inactivated form) after 36 h (Figure-1b), and high levels (710 Lf/mL) of beta toxin using *C. perfringens* type C (NCTC 3180) were obtained. The inactivated beta toxoid was then emulsified 1:1 with an aluminum hydroxide suspension (3%; pH=6.8–7.2) as an adjuvant and kept at room temperature overnight with constant agitation.

**Table-1:** Inactivation kinetics for *Clostridium perfringens* Type C after cultivation for 24 h.

Titer of *Clostridium perfringens* Type C (CFU/mL)		
Time (hours)	Treated with formaldehyde	No treated with formaldehyde
0	4.1×10^9^	4.1×10^9^
4	2.8×10^8^	1.9×10^10^
8	1.9×10^6^	2.1×10^10^
12	1.7×10^5^	4.4×10^10^
16	2.6×10^3^	2.5×10^10^
20	4.3×10^2^	3×10^10^
24	29	1.7×10^10^
32	13	7.2×10^9^
40	1	2.2×10^9^
48	0	3.5×10^9^

**Table-2:** Inactivation of beta toxin of *Clostridium perfringens* Type A.

Titer of beta toxin (LD_50_/ml)		
Time (hours)	Treated with formaldehyde	No treated with formaldehyde
0	710	710
12	9.71	843
24	1.95	599
36	0	705
48	0	837
72	0	709
96	0	708

A stability study was then performed immediately after manufacturing and after 3, 6, 9, 12, 18, 24, and 30 months by monitoring the physical appearance of the vaccine and sterility in addition to pH for each sample. The vaccine was observed to maintain a yellowish-brownish color with a mean (±SD) pH of 6.96 (±0.03) (range, 6.8–7.1). Moreover, the sterility test revealed no growth under both aerobic and anaerobic conditions, indicating that the formulation was not contaminated.

**Figure-2:**
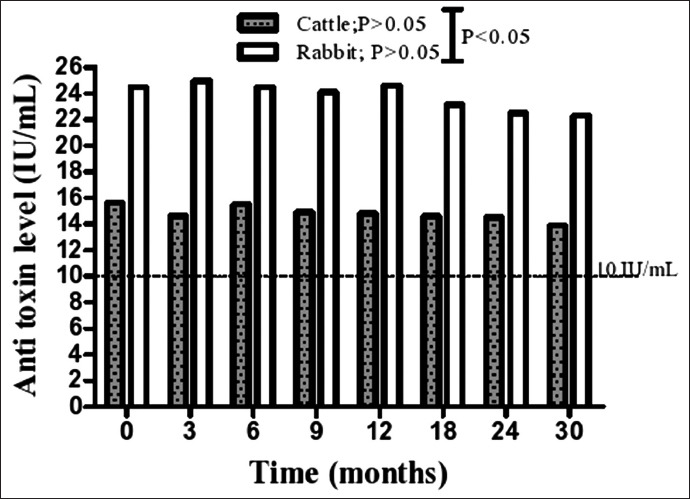
Distribution of anti-toxin levels detected on the potency text in both rabbits and cattle after immunization on using pooled serum, and the dotted line indicate the minimum antitoxin level (10 Ul/mL) suggested by the United States Department of Agriculture. Our findings showed that the developed vaccine was capable of inducing higher immune responses in rabbits than that stimulated in cattle with an extremely significant difference (p<0.0001).

**Figure-3:**
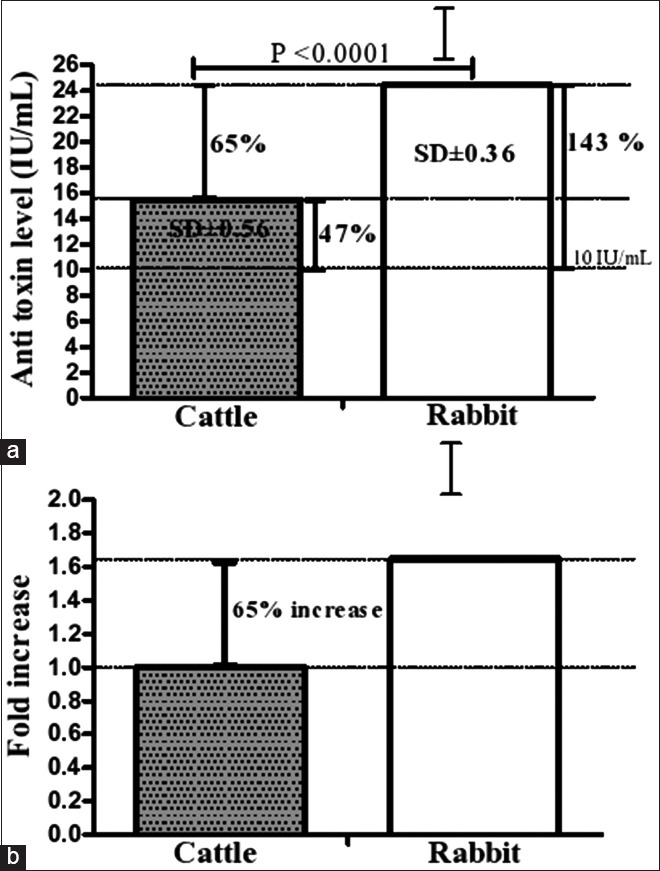
Levels of anti-toxins antibodies on inoculating in different animal models (a) mean values of anti-toxin levels detected in rabbits and cattle after immunization, and (b) distribution of observed fold changes for anti-toxins antibodies in rabbits and cattle. The minimum antitoxin level suggested by the United States Department of Agriculture was 10 Ul/mL.

The production of neutralizing antibodies was measured by seroneutralization studies in mice, and the results of the potency test are shown in Figure-2. Additional target species animals, cattle, were also immunized to verify the results obtained when using rabbits as the model (Figure-2). Our findings showed that our formulation exceeded the minimum antitoxin level (10 IU/mL) established by the USDA [24] since the neutralizing antibody levels produced against beta toxin showed means (±SD) of 24.36 IU/mL (±0.56) and 14.74 IU/mL (±0.36) when using rabbits and cattle as animal models, respectively. For the rabbits, it was estimated that the obtained antibody levels exhibited a 2.43-fold increase (i.e., a 143% increase) relative to the minimum antitoxin level. For cattle, the antitoxin level was inferior to that produced in rabbits, showing a 1.47-fold increase (i.e., a 47% increase) relative to the minimum antitoxin level. Interestingly, our formulation was capable of inducing a 1.65-fold higher immune response in rabbits than in cattle (i.e., a 65% increase); this difference was highly significant (p<0.0001; Figure-3a and b).

To verify the safety of our formulation, three groups of rabbits, including young and adult individuals, were inoculated with the vaccine, and the results are presented in Table-3. The vaccinated young and adult rabbits did not develop any local or general reaction. A non-significant increase in mean rectal temperature was observed over the first 10 h in the vaccinated rabbits (p>0.05). However, the temperature returned to normal after 24 h (Table-3).

**Table-3:** Rectal temperatures after each vaccination in different group of rabbits.

Group	Treatment	Time (hours)							p-value^c^

		24 h before	0 h	10 h	24 h	48 h	72 h	96 h	
Group I^a^	One dose	38.7±0.6	38.8±0.6	39.1±0.6	38.5±0.5	38.8±0.6	38.7±0.6	38.7±0.6	>0.05
Group II^a^	Double dose	38.6±0.7	38.6±0.6	38.9±0.7	38.8±0.7	39.1±0.6	38.6±0.4	38.5±0.4	>0.05
Group III^a^	Repeated dose^b^	38.5±0.5	38.5±0.6	39.0±0.7	38.5±0.5	39.0±0.6	38.6±0.8	38.7±0.5	>0.05
Group IV^a^	One dose	38.5±0.5	38.6±0.6	39.0±0.5	38.6±0.5	38.7±0.5	38.5±0.6	38.4±0.7	>0.05
p value^c^		>0.05	>0.05	>0.05	>0.05	>0.05	>0.05	>0.05	

Continuous variables were expressed as mean±SD.

a Groups I, II, and III are young rabbits; Group IV is adult rabbits.

b Group III was administered an additional dose of vaccine after 3 weeks (21 days).

c p>0.05 is considered non-significant; p<0.05 is considered significant

## Discussion

Protein toxins are important virulence factors of *C*. *perfringens* and play a pivotal role in bacteria surviving and subsequently thriving in an animal or human host. Intoxication by any of these clostridial proteins ultimately leads to cell dysfunction and death [7,25]. Indeed, treatment is usually ineffective due to the severity of the disease. Therefore, vaccination represents the best approach to controlling these infections. Thus, this work was dedicated to producing a newly developed vaccine against the major *C. perfringens* beta toxin that could enhance immune responses in addition to improving the protection in two different animal models. Nevertheless, the factual usefulness of any developed vaccine depends on several factors. First of all, it should be non-pathogenic in animals and have the ability to promote high specific antibody titers. Second, the vaccine should be capable of not only eliciting protective immunity after administration but also being safe, showing the stability of the body temperature without any local or general reaction [26]. Of note, the present work involved simpler procedures and materials for producing a vaccine against beta toxin using *C*. *perfringens* type C (NCTC 3180), which has been proven safe and which produces high levels of beta toxin. Therefore, this process could overcome most of the problems related to the industrial production scale of such vaccines. The described vaccine against beta toxin was found to be non-pathogenic in two very different animal models and had the capability of not only propagating high titers of neutralizing antibodies, being stable and safe, but also eliciting protective immunity after administration and showing thermal stability without any local or general reaction with a feasible quantity. However, the process of the recombinant vaccines against *C. perfringens* beta toxin does not require inactivation after purification; nevertheless, data on the stability of recombinant vaccines are missing. In addition, the process of storing recombinant beta toxin as lyophilized proteins until use and suspending lyophilized proteins in PBS and mixing them with aluminum hydroxide under slight agitation for 20 h at 25°C for proper homogenization and to adsorb the proteins on the aluminum hydroxide surface [27,28] is associated with a high risk of contamination and moreover increases the complexity of the vaccination process and does not give effective results in the field. Furthermore, the *C. perfringens* type C can secrete beta toxin and other types of toxins that cause enteric diseases in various animal species and even other types of diseases; therefore, the vaccine produced from *C. perfringens* C gives immunity to all these types of toxins and proteins. Unfortunately, the recombinant vaccine can only protect from the major beta toxin [7].

Initially, in this study, *C. perfringens* type C, as well as its respective beta toxin, was inactivated by adding formaldehyde as the inactivating agent. This is because formaldehyde, a one-carbon, highly water-soluble aldehyde, has the ability to eliminate any possible harmful effects and make the bacteria unable to replicate or reproduce themselves in addition to detoxifying the bacterial toxins [28,29]. The newly developed vaccine was then formulated and prepared for subsequent evaluation.

The major question then arises as to which extent our developed vaccine would be capable of inducing a relevant immunological response. This question could be answered by noting the level of the produced neutralizing antibodies (24.36 IU/mL), which exceeded the minimum required antitoxin antibody level (10 IU/mL) by 14.36 IU/mL on its inoculating in rabbits. Interestingly, our new formulation was estimated to have a 2.43-fold increase relative to the minimum antitoxin antibody level. The aforementioned findings could indicate that our new formulation might elicit levels of neutralizing antibodies higher than that required by international standards, given the 143% increase.

This work was also dedicated to determining the extent by which the developed vaccine could induce an immunological response not only in rabbits but also in a completely different model, namely, cattle. Indeed, the obtained serology results for cattle appeared less effective than those obtained for rabbits. However, the elicited levels of neutralizing antibodies in cattle were found to be higher than those required by international standards. Interestingly, our newly developed formulation was estimated to provide a 1.47-fold increase over the minimum required antitoxin antibody level in the case of cattle, i.e., a 47% increase. It is noteworthy that our vaccine was more effective in rabbits when compared to cattle in terms of inducing an immunological response. This was evidenced by our statistical analysis, which indicated that the levels of neutralizing antibodies induced in rabbits, which is the animal model for testing *C. perfringens* vaccines, were significantly higher than those obtained in cattle. It was observed that our new formulation showed a 1.65-fold higher immune response in rabbits than in cattle, i.e., a 65% increase, reflecting a highly significant difference (p<0.0001). Consistent to our results, Moreira *et al*. [27] introduced a recombinant vaccine that was effective in generating protective antibodies of 24.4 IU/mL and 13.71 IU/mL in rabbits and cattle, respectively. These results were almost similar to the results obtained in the present work. Surprisingly, and consistent with our results, an approximately 1.69-fold lower immune response occurred in cattle when compared with the rabbit antibody levels observed by Moreira *et al*. [27], which was similar to that produced in our study as mentioned above. In this work, we found that the difference between rabbits and cattle was 10.7 IU/mL and thus similar to that provided previously by Moreira *et al*. [27] for the beta toxin.

Indeed, this study is considered to be the first one published dedicated to investigating the stability and safety of a beta toxin vaccine. It was observed that the vaccine was stable for 30 months without change in potency for both rabbits and cattle. Previously, the stability of the *Clostridium perfringens* type D toxoid vaccine was ascertained after keeping the toxoids at different temperatures, i.e., 25, 35, and 45°C for 45 days [30]. In addition, the developed vaccine was found to exhibit no signs of toxicity or to induce hypersensitivity in any of the animals tested. Our newly developed vaccine has only led to a slight increase in the temperature of tested animals, which returned to normal after 24 h. For vaccine safety evaluation, 2 mL of the *Clostridium perfringens* type D toxoid vaccine was injected intraperitoneally into three healthy rabbits. Neither mortality nor morbidity was recorded for 24-72 h after inoculation [30].

## Conclusion

The research showed a procedure for the manufacturing process of the vaccine against *C. perfringens* beta toxins with a feasible quantity and the vaccine described here showed to be effective in eliciting levels of neutralizing antibodies higher than required by international standards. In addition, The vaccine was stable up to 30 months. Thus, it may represent an effective and safe for preventing *C. perfringens*-related diseases in rabbits and cattle, although further studies to prove its efficacy in the field on other farm animals are still needed.

## Authors’ Contributions

MJS, MFD, and AMA designed the study and drafting the manuscript. MJS, MFD, AMA, and MFB performed all the experimental procedures. MJS and MFD formulated the vaccine and MJS and IJS conducted data analysis and interpretation. All authors read and approved the final version of the manuscript.

## Competing Interests

The authors declare that they have no competing interests.

## Publisher’s Note

Veterinary World remains neutral with regard to jurisdictional claims in published institutional affiliation.
